# Molecular and Seroepidemiological Survey of Visceral Leishmaniasis in Owned Dogs (*Canis familiaris*) in New Foci of Rural Areas of Alborz Province, Central Part of Iran: A Cross-Sectional Study in 2017

**DOI:** 10.18502/jad.v14i1.2702

**Published:** 2020-03-31

**Authors:** Aliehsan Heidari, Mehdi Mohebali, Mozhgan Vahed, Kourosh Kabir, Zabihollah Zarei, Behnaz Akhoundi, Samira Elikaee, Hojatallah Barati, Monireh Sezavar, Hossein Keshavarz, Zahra Kakooei, Homa Hajjaran

**Affiliations:** 1 Department of Parasitology, School of Medicine, Alborz University of Medical Sciences, Karaj, Iran; 2 Department of Medical Parasitology and Mycology, School of Public Health, Tehran University of Medical Sciences, Tehran, Iran; 3 Center for Research of Endemic Parasites of Iran (CREPI), Tehran University of Medical Sciences, Tehran, Iran; 4 Social Determinant of Health Research Center, Alborz University of Medical Sciences, Karaj, Iran; 5 Center of Against Infectious Diseases, Health Depatment, Alborz University of Medical Sciences, Karaj, Iran; 6 Department of Experimental Sciences, Faculty of Allied medicine, Alborz University of Medical Sciences, Karaj, Iran

**Keywords:** *Leishmania infantum*, Dog, Nested-PCR, Direct agglutination test, Iran

## Abstract

**Background:**

Mediterranean form of visceral leishmaniasis (VL) is endemic among some provinces of Iran. The present study was designed to determine the prevalence of canine visceral leishmaniasis (CVL) in the owned dogs of the rural areas of Alborz Province near Tehran as the capital of Iran.

**Methods:**

This study conducted on 303 owned dogs that selected using a stratified random sampling method. The direct agglutination test (DAT) was used to determine the frequency of Vl. The spleen biopsy was taken from the serology-positive dogs for the confirmation of CVL in the suspected dogs. Nested PCR and sequencing methods were used to determine the type of *Leishmania* species in the dogs which were parasitological positive.

**Results:**

Overall, the DAT results of 9 dogs (2.97%, CI: 1.57–5.55) showed anti *Leishmania* antibodies at titers ≥ 1:320 indicating VL infection. One dog (0.33%, CI 95%: 0.06–1.85) showed clinical signs and symptoms of VL. There was a significant correlation between the positive cases of CVL and rural area (p< 0.001). The *Leishmania* was observed in the impression smears that were prepared from spleen biopsy of five the studied dogs. *Leishmania infantum* were confirmed in all them using nested–PCR assay. The sequence analysis of all five isolates was 95% similar to *L. infantum*.

**Conclusion:**

This study shows that domestic cycle of *L. infantum* has been established in rural areas of Alborz province where located near Tehran as capital city of Iran. It is necessary to increase the awareness and monitoring of the disease periodically.

## Introduction

Mediterranean form of visceral leishmaniasis (VL) is an important zoonotic parasitic disease caused by *Leishmania infantum* (1, 2). This form of the disease has been reported from the Mediterranean, three major areas of China, and Brazil (1, 3). This epidemiological form of leishmaniasis is endemic among some provinces of Iran including Ardebil, East Azarbaijan, Fars and Bushehr (4). The disease foci are expanding in other parts of the country, and cases have been reported in other provinces, including the Alborz Province (5). The most reported cases of VL (98%) were found in the rural area of Iran especially in children under 10 years of age (4, 6). Domestic dogs are principal reservoir hosts and source of the infection for humans (7, 8). The causative agent of the disease is transmitted from infected dogs to human by sand fly bite (9).

Identification of infected reservoirs can play an important role in preventing the transmission of *Leishmania* to humans, especially children. Since most infected dogs are clinically asymptomatic, identifying and detecting anti *leishmania* antibodies in sera is appropriate diagnostic method for screening dogs in epidemiological studies and evaluating control programs (10). There are several serological tests for identifying anti leishmania antibodies, among which the direct agglutination test (DAT) is more useful due to its simplicity, no need for special tools, and high reliability and validity (11, 12).

Determining the type of *Leishmania* in VL is important for controlling the disease. PCR-based methods are applied for the isolation and differentiation of *Leishmania* species in infected dogs, especially in areas where both cutaneous and visceral leishmaniasis existed (13, 14). Internal transcribed spacer (ITS) 2 is one of the best parts of the genome to detect closely related species. According to studies, ITS2 has sufficient diversity among different species of *leishmania* parasites and can be used to identify the species (15). Determining the prevalence of CVL especially the owned dogs, which are in more contact with humans and sand flies that feed on them might bite humans, is necessary to adopt an appropriate strategy for controlling the Mediterranean VL (16, 17).

Since there are few studies on the epidemiological characteristics of VL in owned dogs in Alborz Province, and considering the importance of the disease in Iran and reported sporadic cases of CVL in recent years from Alborz Province, and in order to find local foci of the infection in high risk areas of the province, the present study was designed using the DAT serological test, parasitology and molecular PCR methods to determine the prevalence of CVL in the owned dogs of the rural areas of Alborz Province near Tehran as the capital of Iran.

## Materials and Methods

**Fig. 1.**
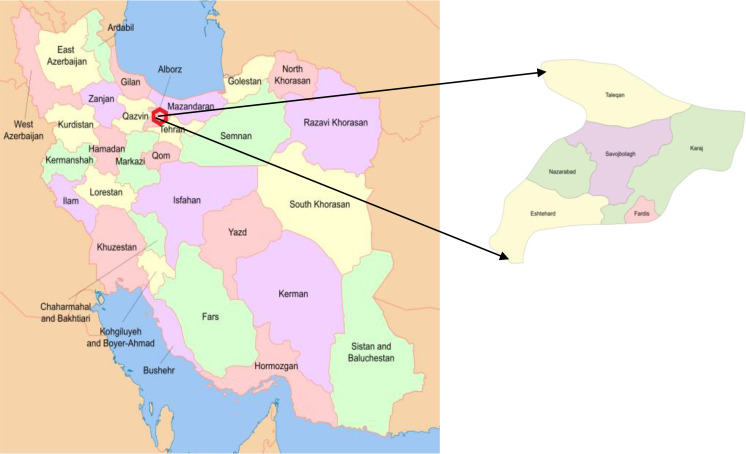
Map of Alborz Province, Iran

Alborz Province is located in the Alborz mountain range slopes. Its area is 833.5km^2^ and its population is 2,712,000 people (18). Nazarabad, Savojbolagh, Taleghan, Eshtehard and Mahdasht, and Karaj-Chalus road are the main rural areas of the province (Fig. 1). Alborz Province is located 30 kilometers from Tehran, the capital city of Iran. The climate of Alborz Province is to some extent cooler than Tehran’s, and it receives 260mm of rain annually.

This study was a cross-sectional descriptive study conducted in 2017 on 303 owned dogs in rural areas of Alborz Province. According to the information of the Veterinary Organization, there were 6560 dogs with collars in the province. The five main rural regions of Alborz Province were considered as strata and a stratified random sampling method was used for the selection of the dogs. The samples were selected randomly based on the number of dogs with collars in each stratum. DAT was applied to determine the frequency of infection with *L. infantum*. PCR and sequencing methods were used to confirm the infection and detect the type of *Leishmania* species in the dogs which were parasitological positive.

Blood samples (3–5cc) were taken from the cephalic or saphenous veins and collected in test tubes. Samples were then transferred to the laboratory of Alborz University of Medical Sciences and centrifuged at about 3000rpm for 15 minutes to separate the serum. Separated serums were stored in a freezer at −20 °C until performing DAT. Frequencies and percentages were calculated, Chi square and Fisher exact tests were used to compare the results.

## DAT test

### Dilution of serum samples of dogs

Serial dilution of serum samples was prepared by adding a 0.9% saline and 0.78% 2-mercaptoethanol. DAT antigen was made from *L. infantum* [MCAN/IR/07/Moheb-gh. [(GenBank accession No. FJ555210)] in RPMI-1640 medium (Biosera, South America) plus 10% fetal calf serum (Biosera, South America), following trypsinization of the parasites, staining with Coomassie brilliant blue R-250 (Sigma, St. Louis, Missouri, USA) and fixing with 1.2% formaldehyde (11, 19).

The antigens were stored in the refrigerator at 4 °C. The antigens then were added to the diluted serums, and the plates were shaken for one minute. The plates were transferred into a damp room and placed on a horizontal surface in the laboratory temperature (22–25 °C) at least for 12–18 hours. The serum sample with the highest dilution that agglutination was produced in was considered as the maximum positive titer for DAT. Positive and negative controls were also used in each test. The presence of an antibody in a dilution equal to or above 1:320 was considered as an infection or disease in the dog.

### Parasitology assay

The spleen biopsy was taken from the serology-positive dogs. The biopsy samples were quickly transferred in the vicinity of spirit lamp flames to several tubes containing the Novy Mac Neal and Nicol and RPMI 1640 culture media. Impression smears were also prepared from the spleen, stained with the Giemsa technique, and examined for the *Leishmania* under a light microscope with high magnification (1000 X).

### Nested-PCR

DNA was extracted from smears stained with Giemsa using the kit protocol of Roche Company (High Pure Template Preparation Kit). ITS2 was used to identify the *Leishmania* species. The following primers were used in this study:

Leish out F (5′- AAA CTC CTC TCT GGT GCT TGC-3′), Leish out R (5′-AAA CAA A GG TTG TCG GGG G-3′), and Leish in F (5′- AAT TCA ACT TCG CGT TGG CC-3′), Leish in R (5′-CCT CTC TTT TTT CTC TGT GC-3′).

PCR was performed according to the method described in the previous study (15).

### Sequencing

Positive samples with Nested-PCR method were sent to the Bioneer Company (South Korea) for sequencing.

### Ethical considerations

Getting a biopsy of the infected dogs was performed according to coordination made with the health care network, environmental and municipal authorities, and observing the ethical considerations of the least pain and suffering. This study was as joint projects approved by the Research Ethical Review Committee of Tehran University of Medical Sciences (Project No.95- 01-160.31439) as well as Alborz University of Medical Sciences, Karaj, Iran with (approval number: Abzums.Rec.1395,8).

## Results

### Direct Agglutination Test

**Fig. 2.**
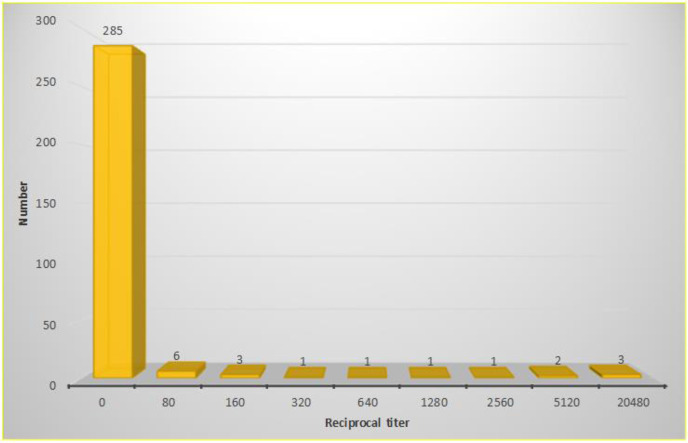
Distribution of Reciprocal titers of anti-*Leishmania* antibodies using DAT among owned dogs of rural areas of Alborz Province in 2017

In this study, 303 dogs of the rural areas of Alborz Province in Iran were tested by DAT method with positive and negative control serum. One dog (0.33%, CI 95%: 0.06–1.85) showed clinical signs and symptoms of CVL. Overall, the DAT results of 9 dogs (2.97%, CI: 1.57–5.55) showed anti *Leishmania* antibodies at titer ≥ 1:320 indicating VL infection (Fig. 2).

In terms of age distribution 194 dogs (64%) belonged to ≤ 3 year-old group and among dogs with anti *Leishmania* antibodies at titers ≥ 1:320, the highest frequency (55.6%) of positive cases was in the 0–3 years age group and the lowest frequency (11.1%) of positive cases was in the > 7 years age group (Table 1). Out of 303 dogs, 254 (83.8%) were male and 49 (16.2%) were female. In terms of sexual distribution, among dogs with anti *Leishmania* antibodies at titers ≥ 1:320, 1 dog (11.1%) was female and 8 dogs (88.9%) were male.

**Table 1.** Frequency of studied dogs in rural areas of Alborz Province in terms of age and sex in 2017

		**Dogs tested (N)**	**Positive**	**95% CI**	**Odds Ratio ((95% CI)**
**sex**	**female**	49	1 (2.04%)	0.36 to 10.69	Male/Female
	**male**	254	8 (3.15%)	1.60 to 6.09	1.56 (0.19 to 12.77)
**age**	**0–3 years**	194	4 (2.06%)	0.80 to 5.18	≥ 4 years/0–3 years
	**≥ 4 years**	109	5 (4.60%)	1.98 to 10.29	2.28 (0.60 to 8.69)
**Total**		303 (100%)	9 (2.97%)	1.57 to 5.55	--------

In terms of distribution of dogs with VL in DAT in rural areas of Alborz Province, the highest frequency of positive cases was observed in Taleghan (Table 2). There was a significant correlation between the positive cases of CVL and rural area (p< 0.001).

**Table 2.** Distribution of positive and negative cases of DAT test in rural areas of Alborz Province in 2017

**Area**	**Dogs tested (N)**	**Positive Antibodies ≥ 1:320 Number (%)**	**95% CI**
**Eshtehard**	75	0 (0%)	-
**Savojbolagh**	76	0 (0%)	-----
**Nazarabad**	64	2 (3.03)	0.83 to 10.39
**Taleghan**	65	7 (10.77)	5.32 to 20.60
**Karaj-Chalus Road**	23	0 (0%)	-----
**Total**	303 (100%)	9 (2.97%)	1.57 to 5.55

A spleen biopsy was taken from 5 of the 9 dogs with anti *Leishmania* antibodies at titers ≥ 1:320 in the DAT test. The *Leishmania* was observed in the impression smears that were prepared from spleen biopsy of five the studied dogs under light microscope with high magnification (1000 X). Examining the culture media, live parasites (promastigotes) were observed in samples of two dogs.

### Molecular results

**Fig. 3.**
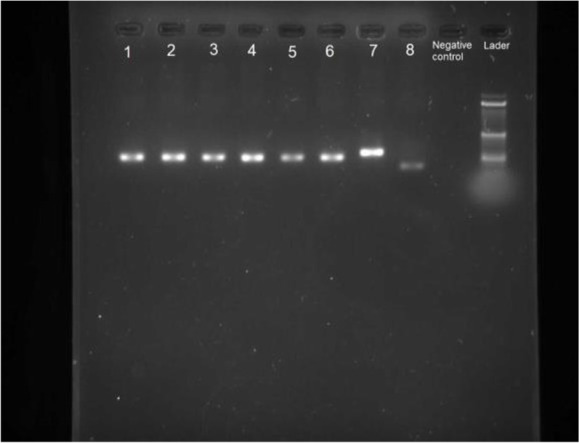
Patterns of ITS2 Nested-PCR products in test isolates and standard *Leishmania* strains. Lanes 1–5: Dogs biopsy samples, lane 6: *L. infantum* (MCAN/IR/07/Moheb-gh), Lane 7: *L. major* (MRHO/IR/75/ER), lane 8: *L. tropica* (MHOM/IR/01/yaza), lane 9: None template control (Negative control), lane 10: Marker (100bp)

DNA was extracted from the positive slides prepared from the spleen of five dogs. *Leishmania infantum* were confirmed in all the five parasitological positive using Nested–PCR assay and 230bp fragment was showed on the agarose gel electrophoresis (Fig. 3). The PCR products were sequenced for species identification.

The sequence of all five isolates was 100% similar to each other and was similar 95% to *L. infantum*. The sequence was recorded in the world gene bank under the MK054276 accession number.

## Discussion

Dogs are the main reservoir of Mediterranean VL. Infected dogs can keep *Leishmania* parasite in their body for a long time and even until death without any symptoms, causing infection of the sand flies (2). From the veterinary and medical point of view, the identification of infected dogs is hence essential for the control of VL in dogs and humans, especially children.

In the present study, 3% (9 cases) of owned dogs had anti *Leishmania* antibodies at titers ≥ 1:320 in the DAT test indicate VL infection. This was lower than the 17% of VL reported in a study on 384 serum samples of owned dogs with DAT test in Meshkin-Shahr, Iran (16), and then the 12% *L. infantum* infection reported in the dogs of nomads in Ardabil province of VL endemic area by DAT test in 2002 (20). It seems that the climate and geography differences, the level of sand flies’ activities and their contact with the reservoirs, and the economic and social conditions, including less livestock farming in Alborz Province, are contributing to the difference in infection levels with the northwest of Iran. In a study on humans in Alborz Province, the rate of human infection with VL was lower than endemic areas of northwest and south of Iran, which is consistent with the rate of infection of dogs (5). In the present study, according to the statistical findings, there was no significant relationship between the age and infection rate of CVL. This was consistent with the results of a study in Brazil, which found no relationship between age and infection rates (21). However, some studies have reported the rate of infection in older dogs due to the possibility of more contact with the sand flies (4, 22). There was no statistically significant difference between sex and leishmaniasis in this study, which was consistent with studies in 1992 in Portugal, 1996 in Greece, and 2018 in Brazil (21, 23, 24). In terms of clinical symptoms in dogs studied in this study, only 1 dog (11.1%) was symptomatic, indicating no significant relationship between positive cases and symptomatic dogs, which was consistent with study conducted in Meshkin-Shahr (13%), but not with a study in Croatia, in which 54% of seropositive dogs from 306 dogs studied with DAT test had clinical symptoms (25, 26). It seems that fewer clinical symptoms are observed in dogs infected with VL in Iran, especially in Alborz Province that is consistent with 5 to 10 percent symptomatic infected dogs in endemic regions (4). The absence of VL infection symptoms in dogs is significant epidemiologically because infected dogs can easily transmit *leishmania* to sand flies (1). In other hand, even treated symptomatic dogs are not cleared from *L. infantum* and can transmit the infection (1, 12). Therefore, regular examination of dogs in CVL high risk areas is necessary.

Determining *Leishmania* species is involved in the adoption of the strategies for preventing, controlling and treatment CVL. Epidemiological evidences and clinical features alone are not sufficient to differentiate the species causing CVL. Parasitology examinations and the use of molecular techniques are hence applied for detecting species and even strains of *leishmania* parasite. Based on the results of Nested-PCR and sequencing of the PCR products, it was found that the disease agent in all five autopsied dogs was *L. infantum*, which is consistent with other studies conducted in Iran (4, 27). Sequencing results provided general view, although limited, of the genetic status of the species causing VL in the studied regions.

Since slides prepared from a biopsy of bone marrow and lymph nodes in dogs with VL, especially in asymptomatic dogs, have a low sensitivity for diagnosis (2), spleen biopsy was used for microscopic examination. In this study, the findings of the microscopic slide examination were consistent with the results of the molecular and serologic tests. This suggests that in the biopsy of the dog’s spleen, the probability of observing the parasite is very high.

Compared with invasive and high risk methods such as taking biopsy from visceral organs including the spleen, liver, lymph nodes and bone marrow, serological methods can be a proper alternative method for diagnosis of CVL (22). Since the disease is prevalent in rural areas with minimum health facilities, it is important to use simple, inexpensive, and reliable diagnostic methods for CVL. Therefore, although DAT was developed several years ago for the laboratory diagnosis of VL, it is still known as the best, most economical and easiest way to detect the disease in dogs.

The geoghraphical distribution pattern of VL infection in dogs was consistent with its distribution pattern among children in Alborz Province. In a study on children under the age of 10, VL cases were observed in Nazarabad and Taleghan. The present study found cases of infection of dogs in these two regions, too. Based on the available evidence and overlap of VL in humans and dogs, and the presence of infected carriers, it seems that Taleghan and Nazarabad are endemic foci of VL in the Alborz Province. CVL was previously reported in Koohsar, a region near Taleghan too (28). The spatial overlap between human and dog visceral leishmaniasis also reprted in Spain (8). The information of this study can help health and treatment planners and managers to prevent and control the infection in the main reservoir of the infection.

In conclusion this study shows that domestic cycle of *L. infantum* has been established in rural areas of Alborz Province where located near Tehran as capital city of Iran.

The high population of dogs in the Alborz slopes and the high population of humans in the province that live the near of the dogs, the infection of about 3% of dogs with the *L. infantum* parasite and the presence of sand flies in the region have provided the condition for transmission the parasite between dogs and humans. Therefore, it is necessary to increase the awareness and monitoring of the infection in dogs periodically. Health authorities should also plan to prevent and control CVL in the Alborz Province.
